# Supplementary material for: MetaRibo-Seq measures translation in microbiomes
Source: Nat Commun. 2020 Jun 29;11:3268. doi: 10.1038/s41467-020-17081-z (PMC7324362; doi:10.1038/s41467-020-17081-z)
Supplement: Supplementary file 10 — Supplementary Data 7 [file 41467_2020_17081_MOESM10_ESM.zip › File2/Confidence_High_Taxonomy/220685_out.krona.html]

Javascript must be enabled to view this page.

members
magnitude
magnitudeUnassigned
count
unassigned
taxon
rank

220685\_out

274

superkingdom
2
274

phylum
273
976

class
200643
273

171549
273
order

family
7
815

genus
816
7

28116
1

SRS144183\_contig\_number\_34667
species

46506
2

SRS014412\_contig\_number\_12934SRS020394\_contig\_number\_17169
species

species

SRS042628\_contig\_number\_contig-100\_71.190097
1
28111

1
329854
species

SRS893369\_contig\_number\_8648

1
47678
species

SRS143523\_contig\_number\_1205


SRS045244\_contig\_number\_4896
species
674529
1

1
171552
family

genus
1
838

1
59823

SRS1041145\_contig\_number\_27223
species

family
171550
265

265
12
genus
239759

SRS015590\_contig\_number\_contig-100\_31579.31580SRS015960\_contig\_number\_20817SRS016267\_contig\_number\_12930SRS016495\_contig\_number\_15665SRS024140\_contig\_number\_contig-100\_11772.11772SRS046502\_contig\_number\_6852SRS063370\_contig\_number\_13418SRS064557\_contig\_number\_contig-100\_1322.116339SRS104311\_contig\_number\_21635SRS142923\_contig\_number\_contig-100\_1743.122314SRS146764\_contig\_number\_7773SRS893327\_contig\_number\_11340

species

SRS011134\_contig\_number\_51569SRS011302\_contig\_number\_15635SRS012849\_contig\_number\_12171SRS012969\_contig\_number\_36532SRS013476\_contig\_number\_contig-100\_1727.199863SRS013521\_contig\_number\_269SRS013940\_contig\_number\_4464SRS013951\_contig\_number\_29838SRS013965\_contig\_number\_24009SRS014235\_contig\_number\_40790SRS014287\_contig\_number\_20065SRS014415\_contig\_number\_1199SRS014459\_contig\_number\_20037SRS014613\_contig\_number\_14004SRS014683\_contig\_number\_18796SRS014923\_contig\_number\_34441SRS014948\_contig\_number\_16761SRS015065\_contig\_number\_25409SRS015264\_contig\_number\_143SRS015782\_contig\_number\_contig-100\_2171.224124SRS015794\_contig\_number\_7181SRS015890\_contig\_number\_3554SRS016018\_contig\_number\_876SRS016056\_contig\_number\_2780SRS016381\_contig\_number\_1290SRS016897\_contig\_number\_355SRS017247\_contig\_number\_10188SRS017307\_contig\_number\_15852SRS017622\_contig\_number\_contig-100\_57.58SRS017745\_contig\_number\_694SRS017859\_contig\_number\_821SRS018133\_contig\_number\_3537SRS018427\_contig\_number\_22185SRS018623\_contig\_number\_13463SRS018836\_contig\_number\_15234SRS018888\_contig\_number\_1803SRS018936\_contig\_number\_9208SRS018984\_contig\_number\_25727SRS019030\_contig\_number\_13881SRS019161\_contig\_number\_19368SRS019178\_contig\_number\_2259SRS019267\_contig\_number\_2475SRS019285\_contig\_number\_33SRS019286\_contig\_number\_6048SRS019381\_contig\_number\_640SRS019496\_contig\_number\_10636SRS019685\_contig\_number\_41814SRS019693\_contig\_number\_6929SRS019808\_contig\_number\_18095SRS019910\_contig\_number\_24643SRS019968\_contig\_number\_1098SRS020233\_contig\_number\_4851SRS020271\_contig\_number\_3886SRS020508\_contig\_number\_31SRS020622\_contig\_number\_397SRS021219\_contig\_number\_3712SRS021484\_contig\_number\_2407SRS021948\_contig\_number\_9699SRS022071\_contig\_number\_23963SRS022524\_contig\_number\_15964SRS023526\_contig\_number\_11913SRS023583\_contig\_number\_3344SRS023715\_contig\_number\_14125SRS023971\_contig\_number\_4222SRS024075\_contig\_number\_9447SRS024132\_contig\_number\_contig-100\_1918.262561SRS024331\_contig\_number\_contig-100\_1608.181800SRS024492\_contig\_number\_10490SRS024625\_contig\_number\_contig-100\_946.97874SRS042966\_contig\_number\_11856SRS043701\_contig\_number\_10675SRS043768\_contig\_number\_24458SRS045004\_contig\_number\_16233SRS045195\_contig\_number\_1245SRS045645\_contig\_number\_30978SRS045713\_contig\_number\_contig-100\_1596.108980SRS046717\_contig\_number\_2700SRS047044\_contig\_number\_16794SRS048060\_contig\_number\_504SRS048870\_contig\_number\_18030SRS049896\_contig\_number\_30599SRS050026\_contig\_number\_1994SRS050299\_contig\_number\_14493SRS050520\_contig\_number\_608SRS050801\_contig\_number\_1015SRS050925\_contig\_number\_17988SRS050998\_contig\_number\_15145SRS051882\_contig\_number\_21060SRS052576\_contig\_number\_contig-100\_481.482SRS053087\_contig\_number\_contig-100\_105.44551SRS053214\_contig\_number\_3124SRS053335\_contig\_number\_11583SRS053649\_contig\_number\_contig-100\_2542.129211SRS054059\_contig\_number\_5166SRS054352\_contig\_number\_829SRS054905\_contig\_number\_4267SRS055017\_contig\_number\_8217SRS055982\_contig\_number\_14276SRS056259\_contig\_number\_30640SRS057480\_contig\_number\_9680SRS058145\_contig\_number\_658SRS062427\_contig\_number\_10472SRS062701\_contig\_number\_5987SRS063040\_contig\_number\_15602SRS063190\_contig\_number\_11488SRS063489\_contig\_number\_5641SRS063518\_contig\_number\_24305SRS063813\_contig\_number\_5438SRS063928\_contig\_number\_86SRS063985\_contig\_number\_13732SRS064757\_contig\_number\_17262SRS065176\_contig\_number\_2403SRS075341\_contig\_number\_1142SRS075821\_contig\_number\_1337SRS076804\_contig\_number\_3730SRS076976\_contig\_number\_15715SRS077024\_contig\_number\_14924SRS077086\_contig\_number\_13314SRS077294\_contig\_number\_19314SRS077392\_contig\_number\_42378SRS077641\_contig\_number\_13121SRS077730\_contig\_number\_9853SRS077849\_contig\_number\_10036SRS078176\_contig\_number\_21865SRS078242\_contig\_number\_2668SRS078419\_contig\_number\_14593SRS078665\_contig\_number\_2886SRS097920\_contig\_number\_2116SRS097958\_contig\_number\_13311SRS098061\_contig\_number\_11323SRS098571\_contig\_number\_13881SRS098644\_contig\_number\_contig-100\_1037.250858SRS100907\_contig\_number\_6031SRS103987\_contig\_number\_4962SRS104036\_contig\_number\_7270SRS1041032\_contig\_number\_8852SRS1041037\_contig\_number\_11065SRS1041039\_contig\_number\_2679SRS1041118\_contig\_number\_10408SRS1041132\_contig\_number\_4839SRS1041133\_contig\_number\_10101SRS1041137\_contig\_number\_2285SRS1041141\_contig\_number\_13011SRS1041143\_contig\_number\_3584SRS104327\_contig\_number\_1941SRS104636\_contig\_number\_21061SRS104693\_contig\_number\_6015SRS104912\_contig\_number\_3984SRS104975\_contig\_number\_12942SRS105153\_contig\_number\_16003SRS1054691\_contig\_number\_9846SRS1054716\_contig\_number\_11556SRS1055022\_contig\_number\_4162SRS1055034\_contig\_number\_3620SRS1055043\_contig\_number\_10058SRS1055067\_contig\_number\_14579SRS1055076\_contig\_number\_11771SRS1055099\_contig\_number\_774SRS142542\_contig\_number\_1107SRS142599\_contig\_number\_13565SRS143148\_contig\_number\_8371SRS143342\_contig\_number\_9741SRS143876\_contig\_number\_2794SRS143895\_contig\_number\_contig-100\_2698.243339SRS144135\_contig\_number\_26060SRS144362\_contig\_number\_11817SRS144603\_contig\_number\_1602SRS144714\_contig\_number\_8123SRS145308\_contig\_number\_5001SRS145497\_contig\_number\_2944SRS147425\_contig\_number\_5020SRS147557\_contig\_number\_32447SRS147766\_contig\_number\_18314SRS148196\_contig\_number\_42673SRS148253\_contig\_number\_9458SRS148424\_contig\_number\_17111SRS148511\_contig\_number\_4234SRS148817\_contig\_number\_3058SRS148874\_contig\_number\_16527SRS149075\_contig\_number\_12944SRS149784\_contig\_number\_27741SRS150029\_contig\_number\_13534SRS893170\_contig\_number\_10837SRS893172\_contig\_number\_10541SRS893187\_contig\_number\_12358SRS893230\_contig\_number\_8355SRS893231\_contig\_number\_1167SRS893279\_contig\_number\_17217SRS893342\_contig\_number\_3303SRS893358\_contig\_number\_10757SRS893373\_contig\_number\_22092SRS893378\_contig\_number\_12832SRS893380\_contig\_number\_483SRS893382\_contig\_number\_3407SRS893383\_contig\_number\_contig-100\_418.172082
28117
195

species

SRS014979\_contig\_number\_contig-100\_1876.186866SRS024388\_contig\_number\_9898SRS065504\_contig\_number\_29199SRS077231\_contig\_number\_3067SRS893341\_contig\_number\_15884
214856
5


SRS064276\_contig\_number\_6824
species
1
1262694

328814
1

SRS144297\_contig\_number\_14441
species

3
1896969
species

SRS019787\_contig\_number\_contig-100\_297.72640SRS074670\_contig\_number\_630SRS142503\_contig\_number\_43140

species

SRS019445\_contig\_number\_24255
1118061
1

1872444
47

SRS011084\_contig\_number\_36028SRS015431\_contig\_number\_63761SRS015663\_contig\_number\_17440SRS016095\_contig\_number\_28988SRS016203\_contig\_number\_8985SRS016954\_contig\_number\_22007SRS017191\_contig\_number\_14923SRS017433\_contig\_number\_17745SRS017821\_contig\_number\_contig-100\_787.173551SRS018817\_contig\_number\_17250SRS019068\_contig\_number\_22736SRS022609\_contig\_number\_40311SRS024435\_contig\_number\_36387SRS043841\_contig\_number\_3841SRS044535\_contig\_number\_14068SRS046068\_contig\_number\_2970SRS046369\_contig\_number\_9928SRS047433\_contig\_number\_13501SRS048164\_contig\_number\_28846SRS049773\_contig\_number\_contig-100\_1553.188097SRS050422\_contig\_number\_contig-100\_1264.168818SRS051031\_contig\_number\_35133SRS053398\_contig\_number\_4380SRS058770\_contig\_number\_contig-100\_1027.140426SRS065397\_contig\_number\_8988SRS075773\_contig\_number\_contig-100\_1684.211479SRS075984\_contig\_number\_contig-100\_1488.144330SRS076756\_contig\_number\_19408SRS098881\_contig\_number\_2856SRS101433\_contig\_number\_12736SRS1041130\_contig\_number\_4064SRS104400\_contig\_number\_12014SRS1055056\_contig\_number\_11816SRS1055083\_contig\_number\_8932SRS142890\_contig\_number\_5036SRS143070\_contig\_number\_11919SRS143181\_contig\_number\_14448SRS143372\_contig\_number\_8778SRS143417\_contig\_number\_16336SRS146812\_contig\_number\_29241SRS147139\_contig\_number\_34068SRS147346\_contig\_number\_61458SRS148091\_contig\_number\_3870SRS892882\_contig\_number\_16276SRS893252\_contig\_number\_18915SRS893366\_contig\_number\_8904SRS971276\_contig\_number\_24411
species

phylum
74201
1

1
203494
class

order
48461
1

family
1647988
1

239934
1
genus

1
239935

SRS1041142\_contig\_number\_7208
species
